# Restoring oysters to urban estuaries: Redefining habitat quality for eastern oyster performance near New York City

**DOI:** 10.1371/journal.pone.0207368

**Published:** 2018-11-16

**Authors:** Katherine McFarland, Matthew P. Hare

**Affiliations:** Department of Natural Resources, Cornell University, Ithaca, New York, United States of America; Bigelow Laboratory for Ocean Sciences, UNITED STATES

## Abstract

Restoring and conserving coastal resilience faces increasing challenges under current climate change predictions. Oyster restoration, in particular, faces threats from alterations in precipitation, warming water temperatures, and urbanization of coastlines that dramatically change salinity patterns, foster the proliferation and spread disease, and disrupt habitat connectivity, respectively. New York City (NYC) coastal waters, once home to a booming oyster fishery for eastern oysters (*Crassostrea virginica*), are now nearly devoid of live oyster reefs. Oyster restoration in urban estuaries is motivated by the synergistic ecosystem benefits this native keystone species can deliver. Recent surveys have documented substantial remnant populations of adult oysters in the upper low salinity zone of the Hudson/Raritan Estuary (HRE) near Tarrytown, NY. This study assessed fitness-related performance across the HRE salinity gradient to evaluate habitat suitability on an estuarine scale. Oysters were hatchery-produced from wild, moderate-salinity broodstock, then outplanted for measurement of growth, survival, reproduction and disease prevalence over two years. Survival was generally higher in the lower salinity river sites and in the higher salinity Jamaica Bay sites relative to mesohaline NYC harbor sites. Growth rate was highest in Jamaica Bay and had high variation among other sites. Surprisingly, the highest proportion of individuals with sex-differentiated gametes and the highest average gonad maturation index was found at a low salinity site. Consistent with the advanced gametogenesis measured in experimental animals at low salinity, annual wild recruitment was documented near the low salinity remnant population in each of five monitored years. These results suggest that the remnant HRE oyster population is a robust, self-sustaining population that can be leveraged to support restoration of subpopulations in other parts of the estuary, but further research is required to determine if the mesohaline and near-ocean reaches of the HRE can support the full oyster life cycle.

## Introduction

Estuarine ecosystems are transition zones that provide essential habitat to hundreds of species, yet rank among the most heavily human-impacted ecosystems because of concentrated urbanization and increased impervious surface [1]. It is likely that humans preferentially colonized the biotically richest estuaries where the natural capital helped support the prosperity and growth of modern coastal cities. There was arguably more at stake, i.e. more to lose, in these estuaries and it could be argued on that basis that there is a moral imperative to restore the ecosystem functions of these urban estuaries, first and foremost. Persuasive arguments for doing so have been made on the basis of economics, human well-being, biodiversity, and coupled human-ecological resilience [2,3]. Nonetheless, contemplating this objective is daunting given the seemingly faint ecological heart-beat of these systems, the extensive hardening of shorelines, and the possibly high costs compared with less degraded estuaries. What’s possible in urban estuaries, and how best to accomplish population replenishment?

New York City (NYC) grew over centuries from a colony enjoying one of the largest and most productive temperate estuaries in the world, the Hudson/Raritan Estuary (HRE). It was among the first megacities in the world (10 million people [4]) and now hosts one of Earth’s greatest concentrations of human habitation and economic activity. For these reasons, and because Hurricane Sandy recently highlighted critical vulnerabilities, it is an urban estuary restoration case study of substantial importance and attracting great interest. Interest in the restoration of ecosystem services has been increasing as indicators of water quality have slowly improved since the 1972 passage of the Clean Water Act [5,6]. One of eleven ecosystem services prioritized for targeted restoration in the HRE is a sustainable oyster population [7,8] because this keystone species has been shown to clarify the water through filter feeding, cycle nutrients from the water column to the benthos, provide habitat for hundreds of other species by building continually accreting reefs, and their reefs can help attenuate storm surge [2,9–12]. Although NYC is the historical home of the eastern oysters’ rise to culinary fame [13,14], largely based on local HRE oyster stocks during the colonial period, now this native species (*Crassostrea virginica* (Gmelin)) is nearly extirpated from the HRE. NYC waters have been closed to oyster harvest for more than a century, initially precipitated by a typhus outbreak and maintained to both minimize public health risks and protect legal oyster commerce [14]. The harvest closures are not expected to change, and possibly can simplify restoration of oyster ecosystem services relative to locations where a fishery generates competing objectives [15].

The importance of metapopulations, semi-isolated populations exchanging migrants among different habitat patches, has long been stressed in ecosystem restoration [16]. Metapopulation connectivity as a restoration goal gains greater importance given global climate change predictions for increasingly more variable rainfall and extreme storm events [17,18]. Demographic and evolutionary resilience is limited in a single population compared with the same biomass distributed as a metapopulation [19–21]. This implies that for population restoration to be effective (i.e., achieve a self-sustaining population), it needs to be to scale across an estuary, especially if the life cycle includes a planktonic dispersal phase [16]. For a euryhaline generalist like *C*. *virginica*, this begs the question of which habitats to prioritize and exactly where [22,23] to focus effort for maximum restoration efficacy?

By definition, the dominant habitat transition in estuaries is the gradient from fresh water entering the system upriver to the oceanic salinities at the mouth, with the mesohaline portion of estuaries often having the highest oyster density [24,25]. For oysters, the salinity gradient is positively correlated with pathogen and predator pressures. Potentially devastating oyster pathogens such as *Perkinus marinus* and *Haplosporidium nelsoni* (dermo and MSX disease, respectively) have dramatically expanded their latitudinal range in recent decades due in large part to warming water temperatures [26,27], exerting the greatest host mortality in moderate to high salinities. Of course, the location of ideal mesohaline conditions for oyster performance in an estuary will transiently shift up and down the estuary with seasonal and annual variation in precipitation or drought, and the margins of suitable habitat will be defined by environmental extremes, not the average.

To restore a metapopulation it does not follow, however, that effort should initially focus on the ‘ideal’ mesohaline habitat, especially if that coincides with the greatest urban impacts. Given the temporally and spatially dynamic distribution of habitat quality, it may be more productive to spread restoration effort across a wide range of habitats rather than focus exclusively or even initially on habitat inferred to be “ideal”. This is not to say that there is no useful information in patterns of relative abundance, temporally averaged abiotic factors, or oyster performance measured during portions of the life cycle in an average year. However, each of these indices may poorly reflect full life cycle effects or the potential for metapopulation synergies. In other words, with respect to estuaries and the sedentary euryhaline taxa that inhabit them, “habitat suitability” indices may put too much focus on average tendencies when the principles of metapopulation resilience suggest that habitat quality variance (spatial and temporal) can be equally important for population viability and regulation [28,29]. For example, metapopulations in the Caloosahatchee Estuary of Florida that span a wide range of salinity variation, provide resiliency for oyster reefs on an estuarine scale by supporting rapid repopulation of local habitat patches following extreme mortality events [30]. Application of metapopulation principles in particular regions requires some understanding of the patterns of population connectivity achieved by oyster larval dispersal, and the spatial and temporal variability in post-settlement viability and reproduction. This study focuses on the second pair of objectives and hypothesizes that genotype by environment interactions will preclude identification of one ideal location within the estuary for strong fitness-related performance of oysters.

**Fig 1:**
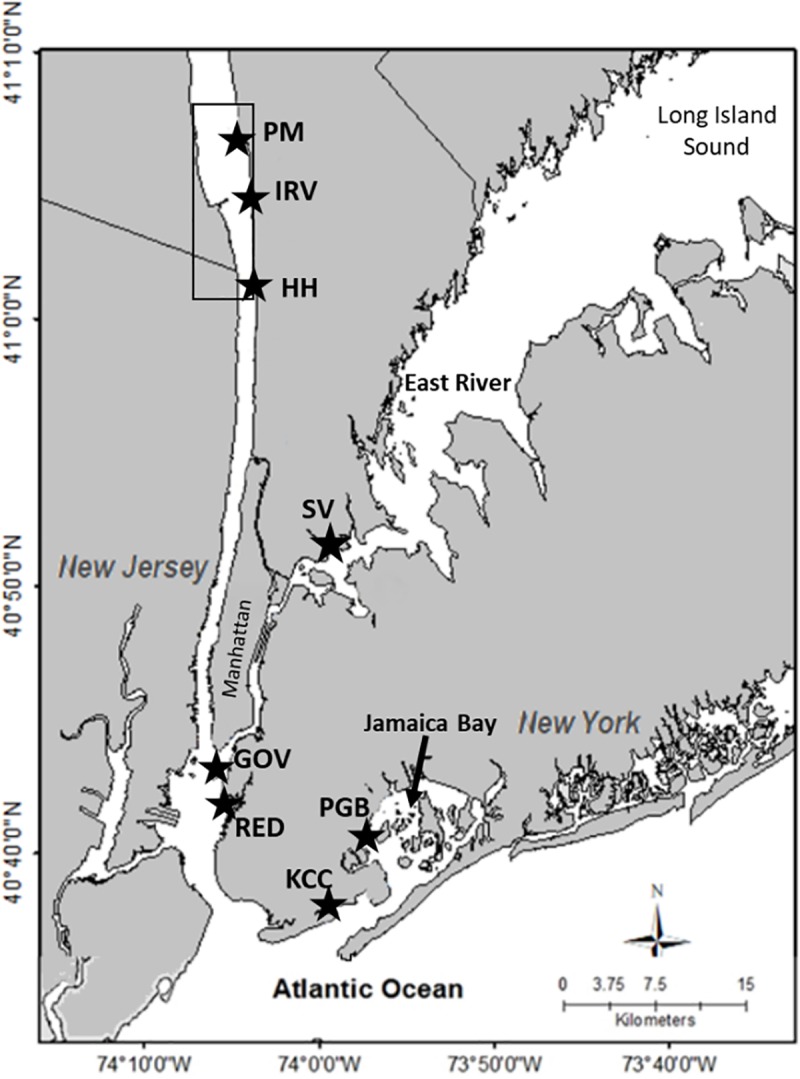
Map of the study sites. Black stars represent outplant cage sites (PM, IRV, HH, GOV, RED, PBG, KCC) and an additional wild spat monitoring site (SV). Sample site names and descriptions are in Table 1. The box represents the Tappan Zee–Haverstraw Bay region supporting a remnant oyster population.

The HRE in New York is a particularly interesting case study for oyster restoration. The mesohaline portions of the estuary, including both the Hudson and East Rivers, currently have no documented self-sustaining reproductive oyster populations, but sparse juvenile (spat) recruitment occurs sporadically [31,32]. The two most proximal potential recruitment sources are Long Island Sound, where Connecticut aquaculture still relies on wild-set oysters [33], and a recently surveyed remnant HRE population in the low-salinity reaches of the Hudson River [29]. Studies conducted by AKRF Inc. on behalf of the New York State Thruway Authority reported mixed-age oyster populations at multiple locations in the vicinity of the Tappan Zee Bridge (now the Governor Mario M. Cuomo Bridge; Fred Jacobs, AKRF, pers. comm.). The remnant oyster population in the Tappan Zee–Haverstraw Bay region (TZ-HB; Fig 1) is often subjected to prolonged salinity exposures below 5 ppt [18]. Previous work in the HRE has documented robust spat recruitment near these remnant populations during years when only sparse recruitment was observed in mesohaline portions of the HRE [18,31,32]. This paper primarily focuses on measuring variation in HRE habitat quality by outplanting an oyster cohort, hatchery-produced from wild mesohaline parents, to measure oyster fitness-related performance along the salinity gradient. Wild-set TZ-HB oysters were opportunistically compared with wild spat from the mesohaline East River and with the experimental cohort at one low-salinity site. Furthermore, we document and compare oyster settlement (spat) recruitment patterns in the HRE over multiple years. Through this work we document regional variation across HRE environments for all major life-cycle stages apart from larvae; spat recruitment, juvenile growth and survival, and reproductive maturation. Given that the only known adult eastern oyster population is restricted to the TZ-HB region, and this implies constraints on down-river population expansion, we hypothesize that one or more aspect of oyster life history will show reduced performance downstream of TZ-HB. Alternatively, urban waters of the New York City harbor may define the region with lowest performance if anthropogenic effects are a dominant limiting factor. Yet another possibility is that oyster performance in experimental cages will be reasonably good everywhere, implicating population constraints stemming from low larval survival/settlement or allee effects. We use the results to consider the feasibility and desirability of seeding restored mesohaline HRE habitat with recruits from the TZ-HB low salinity reefs in order to establish a metapopulation.

## Methods

### Study site and environmental factors

Seven sites (Table 1) were selected for caged oyster outplants, ranging in average annual monthly salinities from 2 to 30 ppt. (Fig 1). We distinguish “river” sites above Manhattan, including Philippse Manor Boat Club (PM), Irvington Canoe Club (IRV) and Hastings-on-Hudson (HH); “harbor” sites near lower Manhattan including Governor’s Island (GOV) and Redhook Waterfront Museum (RED); and two sites in Jamaica Bay, Sebago Canoe Club in Paerdegat Bay (PGB) and Kingsborough Community College (KCC). One additional site in the East River was used to monitor and collect wild spat recruitment (Soundview Park; SV). The East River is the western extension of Long Island Sound and connects with the Hudson River through strong tidal currents at the southern tip of Manhattan. At all sites cages were suspended from docks except HH where cages were secured to the rocky bottom in the intertidal zone with exposure only at extreme low tides. To prevent freezing, HH cages were over-wintered (November–April) subtidally at the adjacent IRV site during 2015–2016, but not during 2016–2017.

**Table 1:** Coordinates for oyster outplant and monitoring sites shown in Fig 1 along with back-up environmental data sources from New York City Department of Environmental Protection.

Site Name	Latitude	Longitude	NYCDEP Station ID	Cage Deployment Type
Philipse Manor (PM)	41.09449	-73.87105		Intertidal hanging fixed depth
Irvinton (IRV)	41.04106	-73.87377		Subtidal hanging fixed depth
Hastings on Hudson (HH)	41.00022	-73.88427		Intertidal benthic
Governors Island (GOV)	40.69137	-74.01252	N1	Subtidal hanging fixed depth
Redhook (RED)	40.67522	-74.01855	G2	Subtidal hanging fixed depth
Kingsborough Community College (KCC)	40.58117	-73.93384	J11	Subtidal hung from floating dock
Sebago Canoe Club (PGB)	40.62645	-73.90456	PB3	Subtidal hung from floating dock
Soundview Park (SV)	40.8105	-73.86606		Spat collection site

At six of the seven monitoring sites (PM, IRV, HH, GOV, RED, KCC) 600 OMS sondes (YSI Inc., Yellow Springs, OH) provided continuous monitoring with hourly measurements of salinity, temperature, depth, and at four sites, dissolved oxygen. During the winter months, all but two sondes (IRV and GOV) were removed due to ice scour risk and oxygen probes were removed from the remaining sondes. Where possible, gaps in the environmental data were filled using nearby data collected by the New York City Department of Environmental Protection (NYCDEP) point readings or our own monthly readings with a handheld salinity/temperature meter. NYCDEP sampling was weekly in the summer and monthly through the winter. All NYCDEP point sampling sites were within 2 km of the applicable oyster cage site. Some environmental data for the IRV river site came from the Piermont Pier HRECOS site (continuous YSI data collection) 2 km directly across the river. All environmental data reported for PGB, and dissolved oxygen for GOV was from point sampling NYCDEP data. During the winter months and for a majority of the 2017 sampling season, all environmental parameters reported for RED, GOV, and KCC also were from NYCDEP to fill gaps due to equipment failure. Only 19% missing covariate data remained after including NYCDEP environmental data.

### Spawning, outplant and phenotyping of oysters

Collection, outplant, and processing of oysters during this study was completed under New York State Department of Environmental Conservation License to collect or Possess (Scientific Permit #1763). Attempts to spawn wild Martha’s Vineyard broodstock failed. Instead, adult F1 oysters from several years of wild broodstock matings, all originating from Lagoon Pond, Martha’s Vineyard, Massachusetts, were strip-spawned in July 2015 at the Martha’s Vineyard Shellfish Group Hatchery (“hatchery cohort” hereafter). Larvae were reared in the hatchery for approximately 2 weeks with no sieve-culling of small size classes and shipped as eye-spot larvae to the Marine and Science Technology (MAST) Center Oyster Hatchery on Governor’s Island, New York (Harbor School, Harbor Foundation) to be set on clean bivalve shell. Spat on shell were initially reared in an upwelling system at the MAST Center (identical to GOV outplant site) until oysters reached approximately 3 mm in length (3 weeks). Average summer salinity of broodstock before spawning was 29 ppt and in the upweller nursery was 24 ppt.

On August 25, 2015, moderate density spat on shell were deployed at the seven HRE sites in 15 mm^2^ polypropylene mesh bags (80–100 shells with spat per bag) in each of three replicate cages per site (2 bags / cage; 3 cages / site). Growth measurements began with the August-September interval. For survival however, reliable spat numbers in each replicate were not counted until September 2015 when spat were culled down to 300 / replicate. Culling was by elimination of more densely-set shells or by randomly thinning spat from such shells. Growth and survival were monitored monthly thereafter with the exception of November–March of each year, and in 2017 no data were collected in April, May and August. The experiment was terminated October of 2017. Length measurements to the nearest millimeter were taken for 100 randomly sampled oysters from each replicate using Mitutoyo digital calipers (Mitutoyo America Co., Aurora, IL, USA). Survival was assessed by counting all live spat in each replicate 2–3 times and averaging the counts. Starting in October 2015, spat ≤ 10 mm were rejected (removed and not counted or measured) to reduce any growth rate or survivorship bias from new wild-set recruits. Post-culling average (SE) spat sizes ranged from 13.3 (±0.84)– 21.1 (±2.7) mm per site at this time. Growth rate estimates were standardized to a 30-day month for comparison between sites and time points. In July 2017, six oyster bag replicates were pooled down to 2 bags at each New York Harbor site due to low survival. Due to overcrowding of cages at Jamaica Bay sites, oysters were thinned down to 100 oysters per replicate in July 2017.

Additional spat on shell from the 2015 hatchery cohort were deployed in cages at each site for destructive sampling. In August 2016 and October 2017 these oysters were sampled to measure condition index (CI). Briefly, individual oysters (N = 25 / site) were cleaned of epiphytic growth, shucked, and soft tissue was dissected from the shell. Both shell and tissue were dried in an oven at 70°C for 48 hours and weighed. Dry weights were used to calculate condition index according to Lucas and Beninger [34] in which higher CI values indicate better physiological condition. For many small oysters, it was not possible to obtain the entire left valve cemented onto the base shell so a modified CI_*rt*_ was calculated for all oysters using only the right (top) shell to allow for comparison among all sites.

$${CI}_{rt}=\frac{dry\;tissue\;weight\;(g)}{dry\;right\;shell\;weight\;(g)}\times 100$$

A second subset of oysters were sampled for *P*. *marinus* infection intensities using Ray’s fluid thioglycolate [35] in August 2016 (N = 10 / site) and July 2017 (N = 20 / site). Briefly, digestive diverticulum sections were dissected and incubated in Ray’s medium for 8 days. Tissue sections were stained with Lugol’s iodine to visualize *P*. *marinus* cells and analyzed microscopically. Infections were scored according to the Mackin Scale [36] from 0 to 5, with 0 indicating no infection and 5 representing heavy infection.

On July 24, 2017, oysters (N = 30 / site) were also sampled for histological analysis to assess reproductive maturity. Briefly, a standardized body cross section was sliced out and preserved in Davidson’s fixative for one week. Tissue sections were then washed in ethanol and processed using standard histological techniques [37] for slide preparation at the Cornell University Animal Health Diagnostic Center. Slides were examined microscopically for gametogenic activity and scored on a scale of 0–10 [38], in which a score of zero was given to oysters with no gonadal activity and oysters ripe to spawn received a score of 5.

### Monitoring wild spat recruitment

Recruitment monitoring was done to learn about the timing and relative abundance of wild spatfall across sites, and also to obtain wild-set oysters for performance comparisons with the hatchery cohort. Recruitment was monitored by deploying 15 mm^2^ polypropylene mesh bags containing clean bivalve shell. Full bags were approximately 30 cm long and 15 cm in diameter. Bags were deployed in pairs at HH and SV in June 2012, 2013, and 2015 and in 2016 and 2017 bags were deployed at HH and IRV. Each month, a minimum of 100 shells were checked for spat settlement and all shells were checked (both sides) if one or more spat was found. If old bags were fouled they were replaced with clean shell bags. In August 2015, wild spat on shell collected at HH and SV were counted, measured and deployed in cages at HH with monthly analysis of growth and survival thereafter.

### Statistical analysis

Normalized growth rate and survival were log transformed to meet the assumptions of normality and heterogeneity and tested for significance using a general linear model with growth or survival being the dependent variable, site and sampling date (month) were fixed factors, cage a random factor and environmental parameters (temperature, salinity, dissolved oxygen) as covariates. Missing environmental data were removed from analysis in a listwise manor (default in SPSS). To better assess variation among seasons and years, survival and growth were calculated separately for summer and winter intervals. To make 2015–2016 over winter survival rates comparable with 2016–2017 they were calculated for an October–June interval. A one-way ANOVA was used to detect differences in interval survival, interval growth, and condition index (August 2016 and October 2017) between sites and years, followed by a Tukey’s HSD post hoc test when differences were detected. The nonparametric Kruskal-Wallis test was used to compare gonad index across sites due to non-normality of the data. All analysis was run in SPSS24 and results represented as means ± standard error to a significance level of *p* < 0.05.

## Results

### Environmental conditions

**Fig 2:**
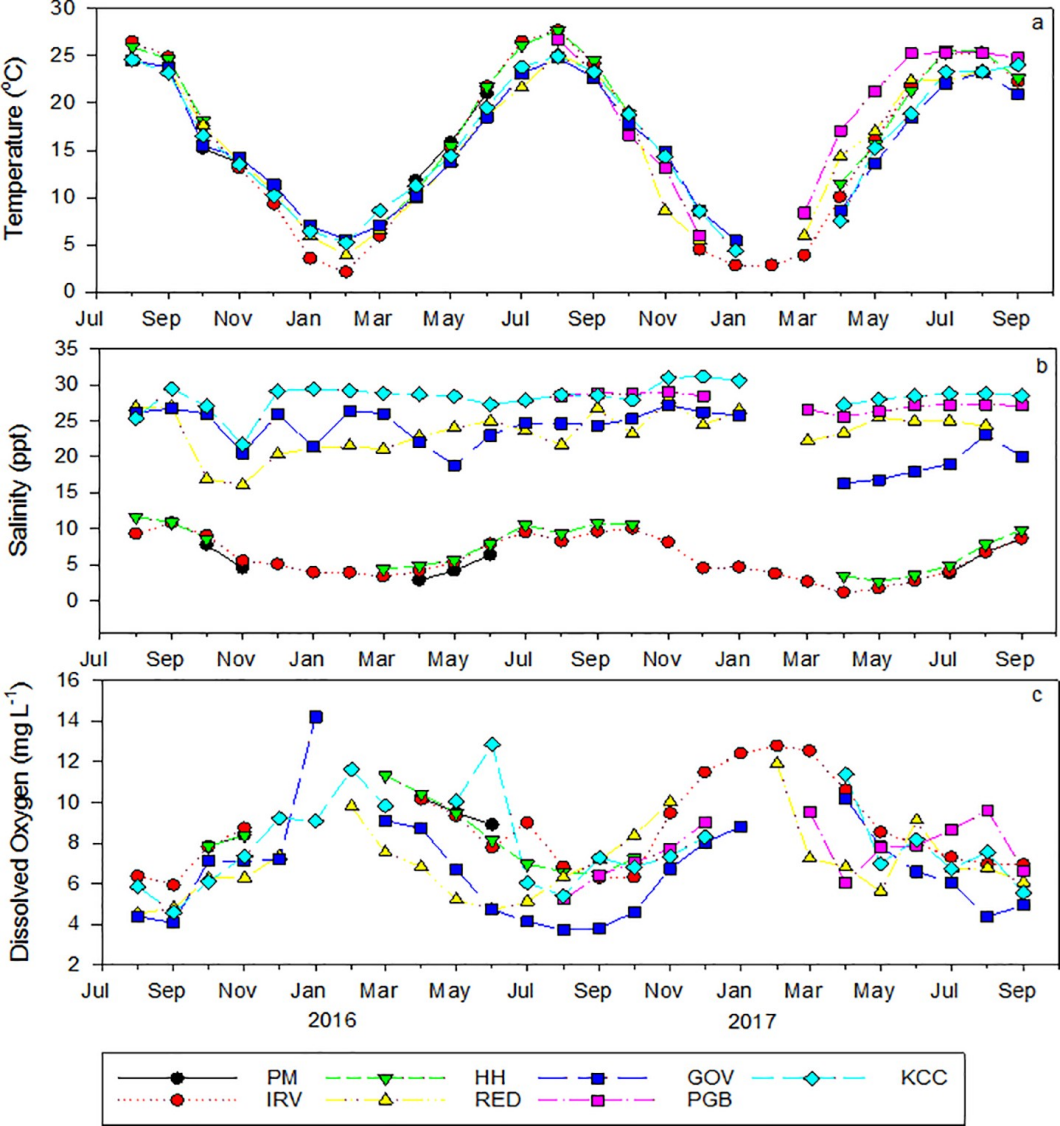
Environmental variables averaged by month for each site. Temperature (a), salinity (b) and dissolved oxygen (c) were monitored hourly at each site. When possible, gaps in data were filled using nearby data collected by the NYCDEP.

Seasonal trends in water temperature were similar among sites and between years (Fig 2A) with daily summer (June–August) maxima ranging 16.8–29.6°C. Salinity showed the greatest variance among sites (Fig 2B) with the most northern sites, referred to here as river sites (PM, IRV, HH), having a range of monthly averages from 1–11 ppt (Fig 2B). The first year of deployment (2015–2016) was a relatively dry year, and although salinities fell below 5 ppt for extended periods of time in the winter and early spring of 2016, mean monthly salinities during the summer (June–August 2016) were 8–10 ppt with daily averages ≥ 5 ppt (S1 Table; Fig 2B). In contrast, high rainfall in 2017 led to significantly lower summer salinities at IRV relative to 2016 (F_2, 52_ = 18.286; *p* < 0.001), with monthly averages remaining < 4 ppt in the spring and through July 2017 (S1 Table; Fig 2B). The two Jamaica Bay sites (KCC and PGB) maintained relatively high salinities, averaging 25–29 ppt throughout the monitoring period. The New York Harbor sites (GOV and RED) had substantial salinity fluctuations, but monthly averages (16–27 ppt) remained within the optimal salinity range for oysters [25].

Monthly averages for dissolved oxygen were lowest in the harbor at RED (≤ 5 mg L^-1^) during the summer of 2016 (Fig 2C; minimum daily average was 2.5 mg L^-1^), with several periods in which the daily average was < 4 mg L^-1^ for 5–14 consecutive days. All other sites had monthly averages ≥ 5 mg L^-1^ throughout the monitoring period.

### Survival and growth

**Fig 3:**
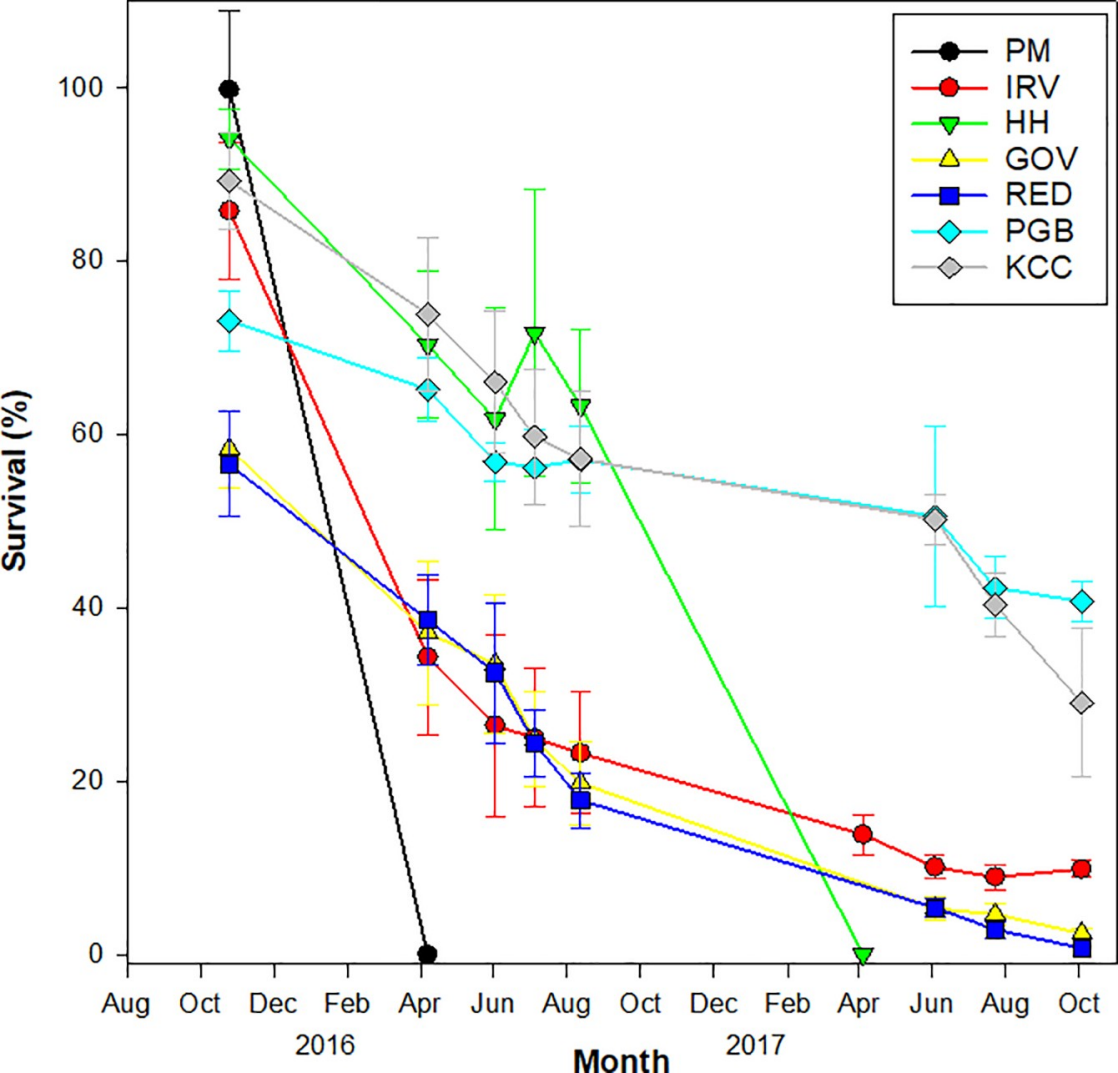
Proportional survival of oysters for each site over time relative to counts in September 2015. Sites are listed in the legend in order of increasing average salinity from the most upstream site (PM) to the most downstream site (KCC). Error bars represent standard error across the 6 replicate cages per site.

Survival varied significantly between sites and over time with a significant interaction between month and site (Table 2). Of the environmental variables measured, only temperature was significantly associated with survival (Table 2). After one month of post-outplant acclimatization, survival from September to October 2015 was high at river sites (86–94%) and Jamaica Bay sites (73–89%), but harbor sites experienced high mortality with only 57–58% survival (Fig 3, S1 Fig). At the end of the first year of deployment (Sept. 2015 –Sept. 2016) survival was relatively high at river site HH (61 ± 9%) and the two Jamaica Bay sites (KCC; 56 ± 8% and PGB; 53 ± 8%) relative to ≤ 20% survival at all other sites (Fig 3). Over-winter survival was very low the first year for two of the river sites, zero survival at PM (the most upstream site) and 41 ± 8% at IRV. Zero survival also was seen at river site HH the following year. Given the cage deployment details at these sites, we suspect cages were exposed to prolonged freezing air temperatures at an extreme low tide and may have played a role in the extreme over winter mortality at these sites. Discounting cases of complete overwinter loss, average overwinter survival was significantly lower than summer survival at river sites (IRV and HH; F_4, 29_ = 8.636; *p* < 0.001 and F_3, 20_ = 2909.895; *p* <0.001, respectively) but not at other sites. Overall, harbor sites had the lowest interval survival during all time points across all sites (Table 3). While river sites had low winter survival, no significant difference in summer interval survival was observed between river and Jamaica Bay sites (Table 3). Survival was low across all sites by the end of the monitoring period with the lowest survival at New York Harbor sites (1% and 2% at RED and GOV, respectively) and highest at Jamaica Bay sites (41% and 29% at PGB and KCC, respectively). River site IRV fell between these two regions with 10% survival after the two-year monitoring period.

**Table 2:** Results of the general linear model of survival (log transformed) and growth rate (log transformed) across sites and time with environmental variables as covariates.

	Source of Variation	Type III Sum of Squares	df	Mean Square	F	Sig.
Survival	Corrected Model	696.395^a^	46	15.139	517.885	<0.001*
	Intercept	0.079	1	0.079	2.707	0.101
	Temperature	0.14	1	0.14	4.794	0.03*
	Salinity	0.001	1	0.001	0.027	0.871
	Dissolved oxygen	0.019	1	0.019	0.656	0.419
	Site	47.965	5	9.593	328.164	<0.001*
	Month	80.708	7	11.53	394.414	<0.001*
	Site * Month	413.994	31	13.355	456.844	<0.001*
	Error	6.577	225	0.029		
	Total	856.835	272			
	Corrected Total	702.972	271			
Growth Rate	Corrected Model	692.949^b^	45	15.399	2.41	<0.001*
	Intercept	6.628	1	6.628	1.037	0.31
	Temperature	32.77	1	32.77	5.129	0.025*
	Salinity	5.306	1	5.306	0.83	0.363
	Dissolved oxygen	2.004	1	2.004	0.314	0.576
	Site	39.436	5	7.887	1.234	0.294
	Month	110.099	7	15.728	2.462	0.019*
	Site * Month	219.003	30	7.3	1.143	0.287
	Error	1412.047	221	6.389		
	Total	2129.418	267			
	Corrected Total	2104.996	266			

Asterisks (*) denotes significance.

a. R^2^ = .991 (Adjusted R^2^ = .989)

b. R^2^ = 0.329 (Adjusted R^2^ = 0.193)

**Table 3:** Mean interval survival for winter and summer periods by year for each site. Due to differences in sampling months between years, intervals were adjusted for best comparison. Letters indicate significant differences among sites for each time interval (i.e., comparable down columns; Tukey’s HSD; *p* < 0.05). Interval survival for RED during summer 2017 was not included in post hoc analysis due to a lack of replicates.

	Mean summer interval survival			Mean winter interval survival	
	2015	2016	2017	2015–16	2016–17
	Sept 27 –Oct 25	June 3 –Aug 16	June 6 –Oct 3	Oct 25 –June 3	Aug 16 –June 6
PM	100%	0%			
IRV	86%^AB^	103% *^A^	99% ^A^	33% ^B^	55% ^AB^
HH	94% ^A^	109% *^A^		67% ^A^	0% ^C^
GOV	58% ^DC^	59% ^B^	42% ^B^	56% ^AB^	30% ^B^
RED	57% ^D^	54% ^B^	14%	57% ^AB^	35% ^B^
PGB	73% ^BC^	98% ^A^	92% ^A^	70% ^A^	83% ^A^
KCC	89% ^A^	86% ^A^	40% ^B^	72% ^A^	75% ^A^

*An increase in numbers was occasionally observed at river sites due to wild spat recruitment, resulting in greater than 100% survival in some cases.

Predators could have contributed to mortality, especially during summer. A heavy presence (> 10 worms observed) of the predatory worm *Stylochus ellipticus* was often observed at RED and GOV with only moderate (5–10 worms) to low (< 5 worms) presence observed at KCC and PGB. *Stylochus ellipticus* was present at river sites in the months immediately following the initial outplant but was not present during other months. The boring sponge *Cliona celata* was observed in summer 2017 at moderate densities at PGB and harbor sites, but high densities at KCC and absent from river sites. Of the 25 randomly sampled oysters dissected for condition index in October 2017 at KCC (see below), one was riddled with *Cliona*, two had initial intrusion to the muscle and all oysters had at least some signs of boring sponge (e.g. holes in the shell and sponge within the shell, but not penetrating the inner layer).

**Fig 4:**
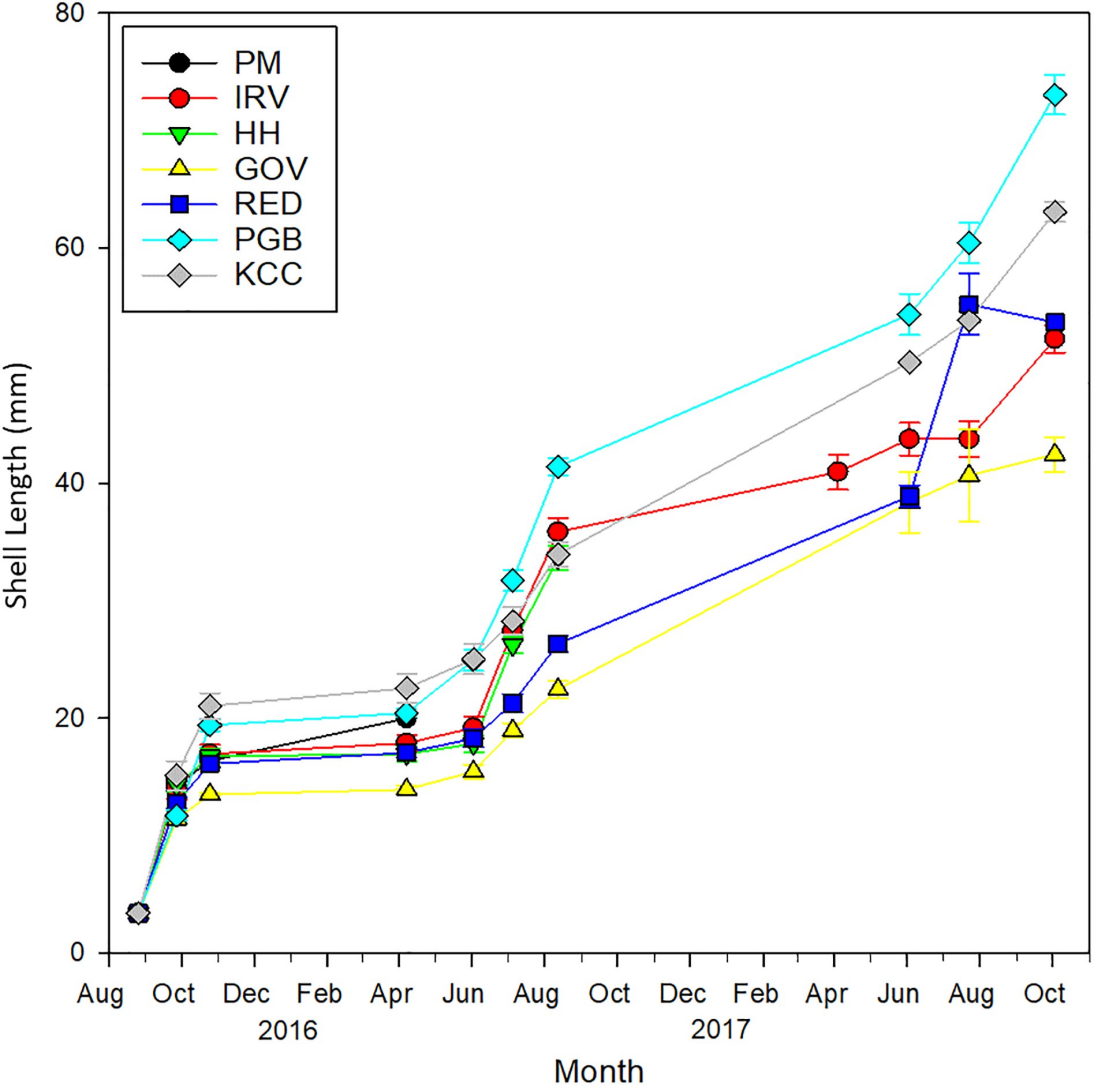
Mean shell length (mm) by site over time. Sites are presented in order of increasing salinity from the most upstream site (PM) to the most downstream high salinity site (KCC). Error bars represent standard error.

Juvenile oyster growth varied significantly by month, but not by site when grouped in the model (Table 2; Fig 4), however when trends were analyzed by interval (one-way ANOVA) significant differences between sites were observed (Table 4). The highest growth rate was observed in the first month of deployment (August–September 2015) ranging from 7–10 mm month^-1^ across all sites. Growth decreased to 2.2–3.6 mm month^-1^ in the second month of deployment (September–October 2015) except at the two Jamaica Bay sites (PBG and KCC) which remained elevated (8.2 ± 0.82 and 6.4 ± 0.63 mm month^-1^, respectively) (Fig 4). No significant growth was observed throughout the winter months (< 0.5 mm; November 2015 –April 2016) and remained near zero between April and June 2016 for all sites except PBG and KCC in Jamaica Bay (0.5–2.1 mm month^-1^). High growth rates resumed in June 2016 and were sustained through August, with the highest monthly averages observed at IRV, HH, and PGB (6.6–7.5 mm month^-1^, 5.8–7.7 mm month^-1^, and 6.2–7.6 mm month^-1^, respectively). Similar growth patterns in 2017 led to cumulative 2-year growth that was highest at PGB in Jamaica Bay (73.05± 1.70 mm) and smallest at GOV in the harbor (42.44 ± 2.56 mm) (Fig 4). Tukey’s post hoc contrast among regions found the higher Jamaica Bay growth rate to be significant, but the harbor and river regions were not significantly different. A spike in growth observed in July 2017 at RED is likely to be artefactual, due in part to size-biased mortality (S2 and S3 Figs; see Discussion) and should be interpreted with caution.

**Table 4:** Mean interval growth in shell length (mm month^-1^) for winter and summer periods by year for each site. Growth rates are standardized to a 30-day month to increase comparability. Due to differences in sampling months between years, intervals were adjusted for best comparison.

	Mean summer interval growth				Mean winter interval growth	
	2015	2016	2017	2016		2017*
	Aug 25 –Oct 25	June 3 –Aug 16	June 6 –Oct 3	Oct 25 –June 3		Aug 16 –June 6
IRV	6.64 ^B^	7.05 ^A^	2.09 ^AB^	0.30 ^AB^		0.80 ^A^
HH	6.55 ^B^	6.68 ^A^		0.14 ^B^		
GOV	4.95 ^C^	2.83 ^C^	1.00 ^B^	0.31 ^AB^		1.62 ^A^
RED	7.08 ^B^	3.40 ^BC^	3.64	0.29 ^AB^		1.28 ^A^
PGB	8.56 ^A^	6.67 ^A^	4.59 ^A^	0.75 ^A^		1.32 ^A^
KCC	8.66 ^A^	3.76 ^B^	3.15 ^A^	0.54 ^A^		1.66 ^A^

Letters indicate significant differences among sites for each time interval when multiple cage replicates remained (i.e., comparable down columns; Tukey’s HSD; *p* < 0.05).

*Elevated growth during the winter 2017 interval is attributed to late growth in the fall (August–October) during which no measurements were taken, but previous years showed this to be a period of high growth. Thus, comparison of growth intervals between years should be interpreted with care.

Growth rate varied significantly with temperature (Table 2) showing a strong seasonal effect. Relatively fast summer growth at low salinity river sites was only observed after spring to summer increases in salinity, contributing to the statistically significant association of growth with salinity. Fastest cumulative growth was observed at Jamaica Bay sites where salinities were consistently high (> 25 ppt). Apparent salinity effects were seen at river site IRV where no growth was observed in July 2017, with average salinity < 4 ppt, yet a high growth rate equivalent to other sites was observed at IRV when salinity increased in August to 6–10 ppt (Fig 4). Dissolved oxygen was lowest at harbor sites in association with the lowest observed growth rates (Fig 4, Table 4).

### Condition and disease

**Fig 5:**
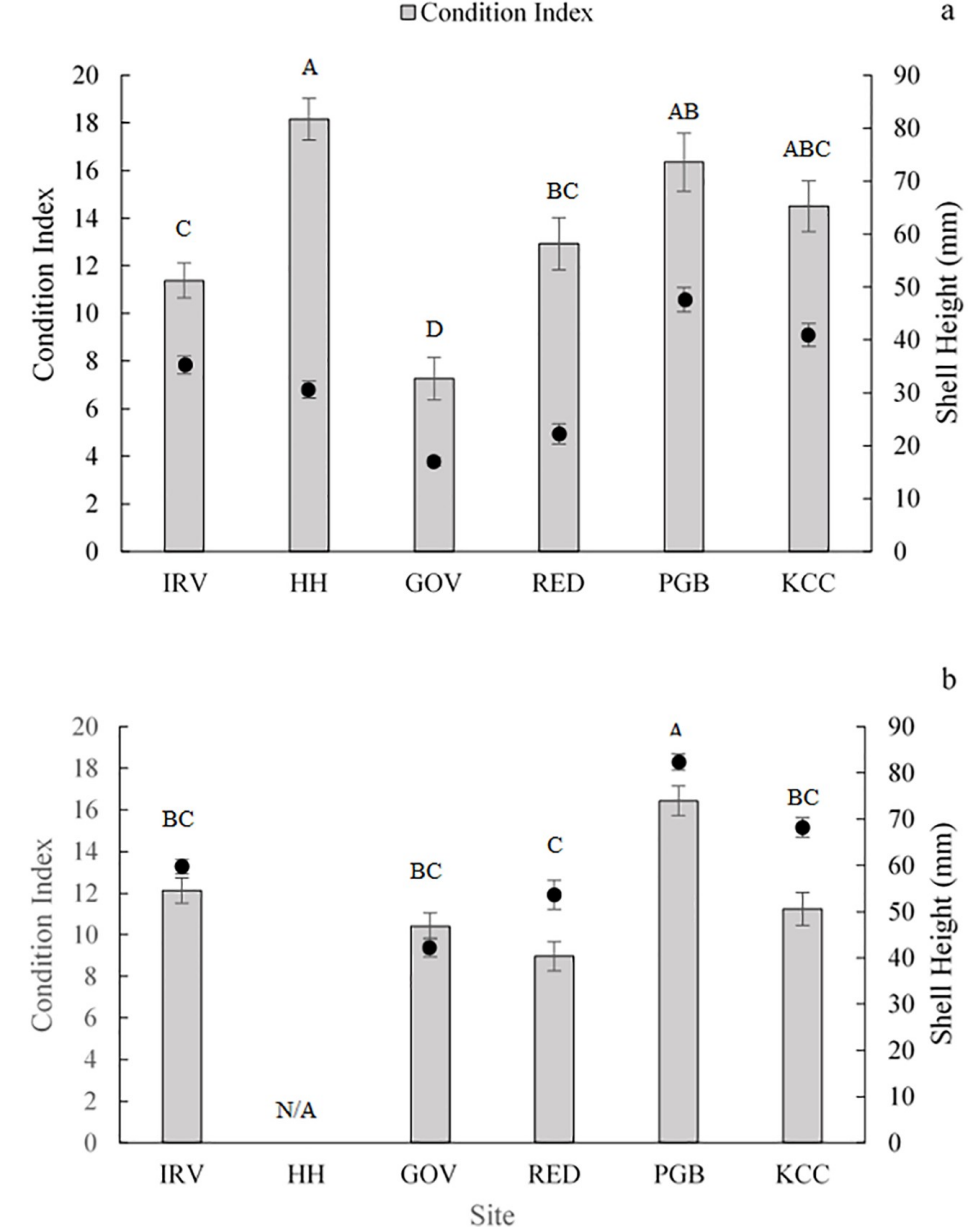
Mean right shell condition index rankings (bars) and mean shell length (points) of all oysters processed for the measurement of condition index at each site after one year of deployment in August 2016 (a) and at the end of the monitoring period in October 2017 (b). Error bars represent standard error. Letters indicate significant difference in condition index between sites (pot hoc Tukey’s HSD *p* < 0.05).

Condition index varied significantly between sites in 2016 (F_5, 137_ = 13.920; *p* < 0.001) and 2017 (F_4, 111_ = 13.949; *p* < 0.001). In 2016, condition index was lowest at IRV and GOV, and highest at HH, PGB and KCC (Fig 5A), similar to trends observed for survival after discounting the complete mortality at HH. Interestingly, in 2016 there was as much variance within river and harbor regions as across all sites. In 2017, PGB had the highest condition index ranking and other sites were similar (Fig 5B). Mean length of oysters sampled in August 2016 was 33.2 ± 1.0 mm and in July 2017 was 56.8 ± 1.4 mm (Fig 5).

Dermo infections were light or absent at all sites during sampling in both 2016 and 2017. In August 2016, three individuals out of 20 at RED and one individual each at KCC and PGB had a single hypnospore, scoring a 0.33 (out of 5) on the Mackin scale. In July 2017, GOV and PGB each had a single individual with an infection rank of 0.33 and RED had 30% prevalence and an average ranking of 0.36 ± 0.13.

### Gametogenesis

**Fig 6:**
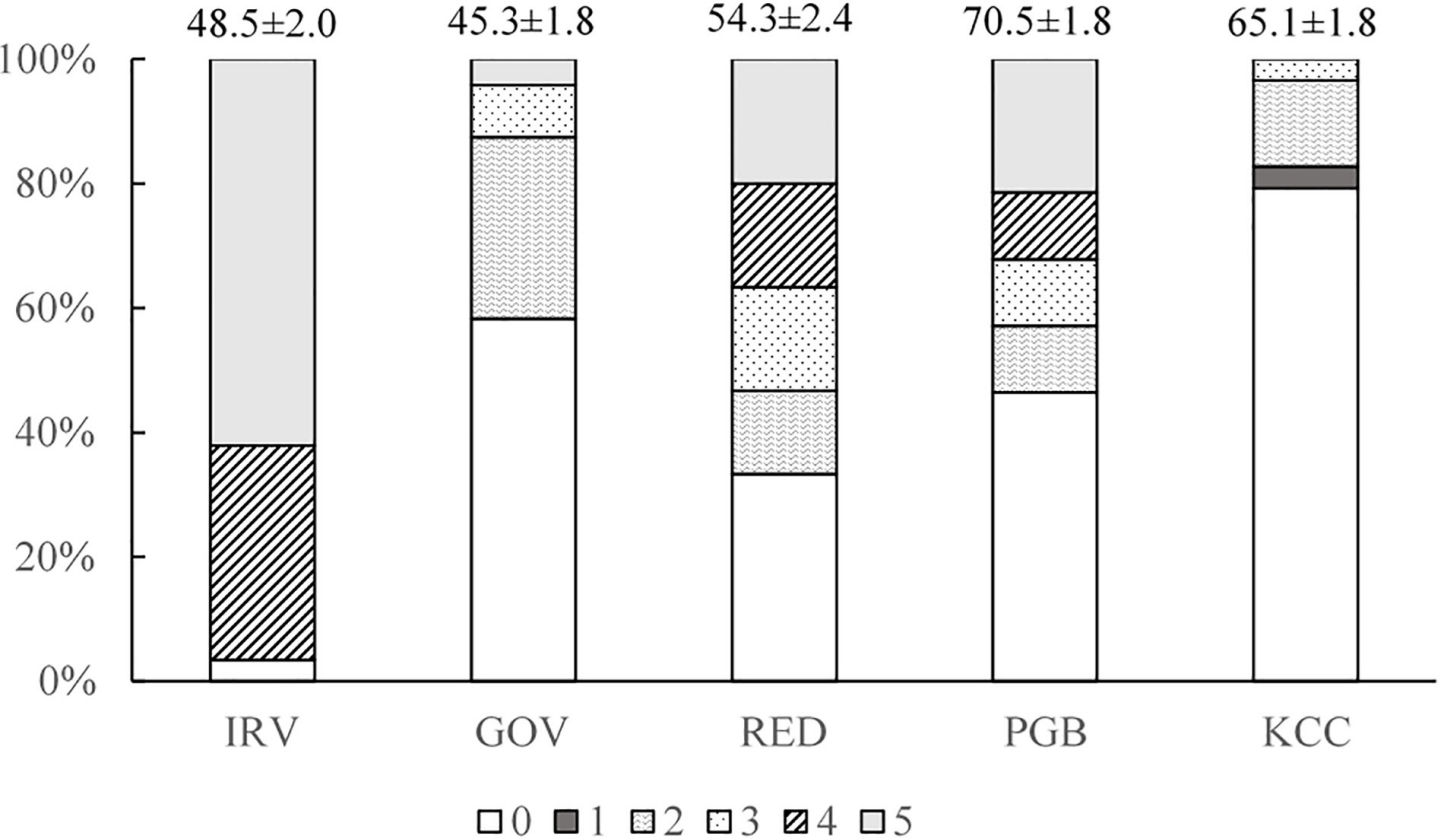
Percentage of individuals (N = 30 / site) at each stage of gonad development in July 2017 at each site. Average oyster size (shell length ± SE, mm) given above each bar.

July 2017 gonad index varied significantly between sites (χ^2^ = 64.047; *p* ≤ 0.001). IRV had the greatest proportion of individuals showing sexual differentiation (97%; categories >0) in which active gametogenesis allowed for the identification of sex, while all others showed a high prevalence of inactive individuals (mean 64.8%), despite significantly larger oyster size in Jamaica Bay (Fig 6). Among individuals with differentiated gamete cells (categories 1–5), the highest gonad maturation index average was observed at IRV (4.6 ± 0.1) and the lowest at KCC (2.0 ± 0.3) (Fig 6). Interestingly, both the harbor and Jamaica Bay regions had one site where a few individuals were in spawning condition, indicating large variance in reproductive conditioning even within regions. Overall, no significant effect of oyster size on gonad index (Chi-square test) was observed among or within sites.

### Wild spat recruitment

Wild spat were consistently observed at all monitored sites (SV, HH and IRV) each year in early fall, except in 2013 when SV yielded no spat in August or September while HH had a successful recruitment year (Table 5). Surprisingly, wild-set spat recruitment occurred at river sites even in 2017 when June daily average salinity never rose above 5 ppt and July only had 10% of days with salinity > 5 ppt (Table 6).

**Table 5:** Mean number of spat per shell bag over the duration of monitoring. Bags were deployed each year in July or August and monitored monthly for new recruits with new clean shell bags deployed each month. No monitoring was completed during 2014; “-” indicates no bags deployed, “0” is zero wild set.

August		2012	2013	2014	2015	2016	2017
	IRV	-	-	- - -	19	25	-
	HH	28	0		34	29	-
	SV	160	0		17	-	-
September							
	IRV	-	-	-	*	160	*
	HH	79	158	-	*	172	108
	SV	23	0	-	-	-	-

* No shell bags were deployed. However, wild spat recruitment was evident from the increased number of spat on shell from August to September when August wild spat on shell was deployed in cages.

**Table 6:** Percentage of days during which the mean daily salinity was > 5 ppt at river site IRV during the summer reproduction and recruitment season. For years prior to sonde deployment (2012–2014), salinity data were obtained from the Hudson River Environmental Conditions Observing System at a site near Irvington (Piermont Pier, NY) from hourly recordings of environmental conditions.

	2012	2013	2014	2015	2016	2017
June	50%		30%	43%	100%	0%
July	100%		23%	32%	100%	10%
August	100%	100%	77%	100%	100%	90%
September	100%	93%	100%	100%	100%	100%
October	83%	100%	100%	100%	100%	100%

**Fig 7:**
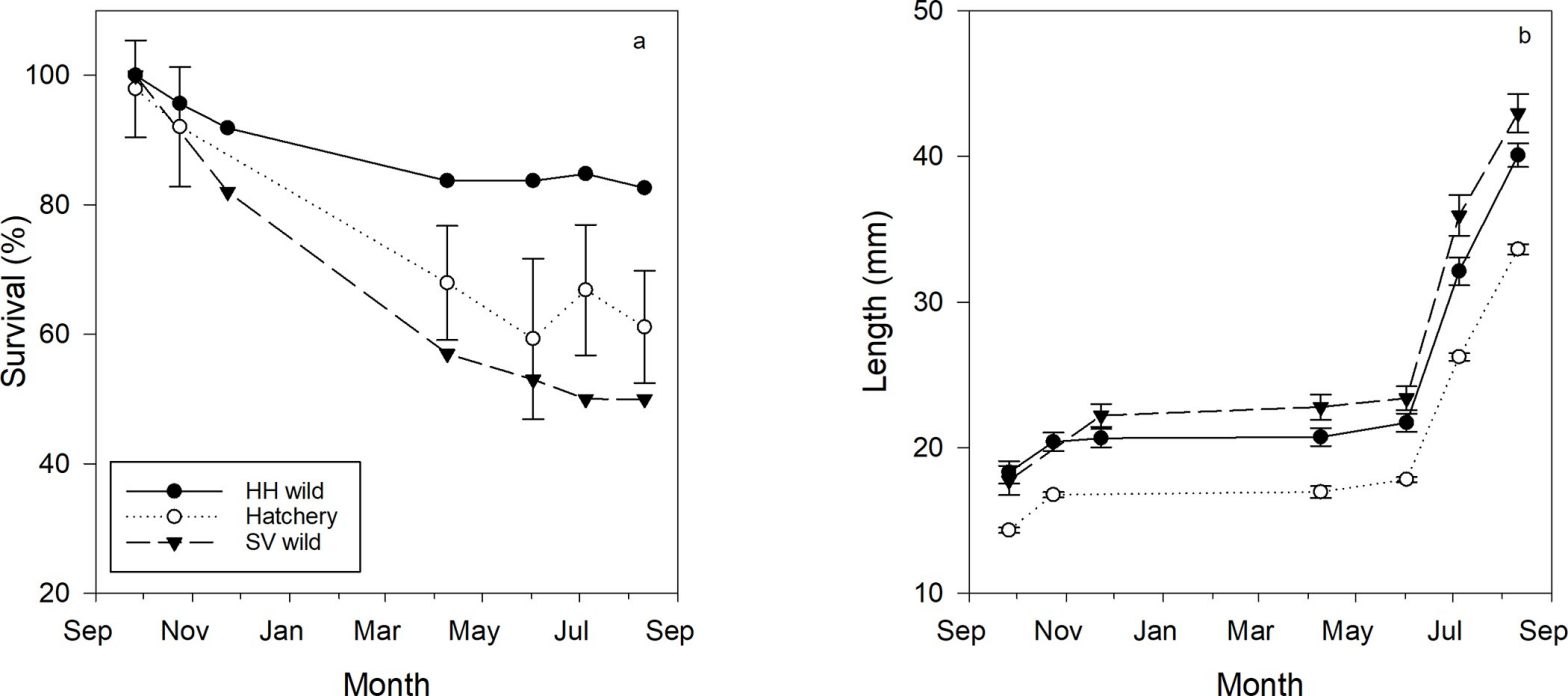
Proportional survival (a) and mean shell length (b) by month from September 2015 for wild spat on shell from HH and SV sites and hatchery produced spat on shell deployed in experimental cages at the HH site.

August 2015 wild-set spat numbered 34 at SV and 68 at HH. After deployment in the HH cages, these initial spat counts increased in September 2015 indicating additional settlement of wild spat. Therefore, September counts were used to initiate wild spat survival monitoring at HH (N = 92 and 48 for HH and SV-mixed sources, respectively). This ‘contamination’ of SV spat with later HH spatfall prevents a strict comparison of performance by spat origin, but the wild spat remained a categorically useful comparison to the hatchery cohort, with one caveat. The wild spat were mixed-age and as much as a month younger than hatchery spat, whereas hatchery spat spent 3 weeks in relatively low growth harbor waters before transplantation to cage sites. Using September 2015 as time zero, after one year of deployment wild HH spat showed a graphical trend for greater survival (83%) compared to both wild SV-mixed spat (50%) and the hatchery cohort at HH (61%) (Fig 7A). Growth rate was similar between wild and hatchery cohorts, so wild oysters retained their initially larger size through the monitoring period (Fig 7B).

## Discussion

A sustainable oyster population requires larval survival, suitable habitat for larval settlement (spat recruitment), some postsettlement survival to maturity, and sufficient fecundity to maintain or grow population size given the other vital rates. Rarely are all components of the oyster life cycle considered in restoration appraisals because reproduction and recruitment are so difficult to measure [39,40], but see [41–44]. Yet strong postsettlement performance yields little restoration gain toward a sustainable population in habitats where gonad maturation is minimal, or where conditions are poor for larval survival. The results of this study inform us about spatial variation in these fitness-related measures of oyster performance, namely post-settlement growth, survival, and gonad maturation at age ~2 years (see also [29]).

Survival to reproduction is an obvious fitness component, but needs to be considered in the context of a life history in which massive early mortality is expected even in healthy populations [45–47]. This species has such a high fecundity that several large females could repopulate an estuary, and a typical year’s cohort will have successful offspring from relatively few females (sweepstakes reproduction [48]). There is massive over-production of larvae followed by high mortality rates during early life history stages in this classic type III survival. Postsettlement survivorship is not only determined by competition and vulnerability to predators [49], in addition to abiotic factors, but also by the Mendelian roulette of inheritance with respect to deleterious alleles carried by parents [50] and the interaction between genotype and microhabitat where an individual settles [51,52]. Thus, to predict the best spatial context for metapopulation recovery it is the relative survivorship among sites, taking into account genotype by environment interactions, that is a key performance measure in addition to absolute survivorship. In oysters the timing and extent of seasonal gonad maturation can identify the age of first reproduction, a parameter of great importance in temperate populations that have one peak reproductive season per year. Oysters that reproduce in their second year of life may have considerably higher average lifetime fitness than those that delay until year 3. This argues for fast juvenile growth to be strongly favored if it accelerates the time to maturity in addition to hypothesized effects on competition for space and protection from predators, but interaction effects with fall fattening and over-winter survival make fitness predictions tenuous. Fast growth has sometimes been assumed to be fitness-enhancing for oysters because it can make for strong space competitors, it can shorten the time to first reproduction, and it makes larger oysters that are generally more fecund [53,54]. However, fast growth may trade-off with hardiness or survival [55], so we only tentatively interpret fast growth as fitness enhancing.

We had three hypotheses for factors determining the spatial arrangement of oyster performance in the Hudson/Raritan Estuary. We caution that two years of data are potentially misleading about population fitness for an organism that can live 20 years, especially given the recent discovery of an 11.5 cm wild oyster on subtidal West Manhattan pilings [56]. The strong performance of Jamaica Bay oysters in terms of growth and survivorship to age 2 might not translate into high fitness if the *Cliona* sponge infestation or other parasites reduced average longevity. We found significant heterogeneity among sites in survival, but not growth, over the first two years of life for a hatchery-produced oyster cohort from mesohaline broodstock. The spatial heterogeneity does not easily align with any of our hypotheses except for survivorship, with harbor sites generally showing the lowest survivorship. For the mesohaline region of HRE to have the lowest survivorship is the inverse of what has been found in other estuaries [24,57,58], where low salinity and community dynamics were blamed for relatively lower survivorship at the upstream and downstream reaches of estuaries, respectively. The high salinity Jamaica Bay sites fairly consistently had the strongest growth and survival, but also had the highest prevalence of parasites and relatively low July gonad maturation. Only the river sites showed a mixture of strong performance, e.g. 2016 summer growth and survival as high or higher than Jamaica Bay, coupled with low disease incidence and a high proportion of nearly-ripe individuals at age 2. Based on late July samples, only caged oysters at the IRV river site showed histological evidence for substantial reproductive capacity at the population level. Strong performance at low salinity is counter to previous comparisons across the salinity gradient in other estuaries. These patterns provide support for the hypothesis that full life-cycle performance is highest in the TZ-HB region where the remnant subtidal oyster population persists. Environmental factors in the harbor, and even in Jamaica Bay, seem to be constraining one or more aspects of oyster performance relative to the TZ-HB region. The environmental factors measured in this study provided no compelling explanatory associations, but the intra-region performance heterogeneity suggests temporally and spatially discreet influences. It is possible that environmental constraints downstream of the TZ-HB region are sufficiently weak or intermittent to allow for a self-sustaining oyster population, if only allee effects could be overcome. However, we consider allee effects to be unlikely as a primary limiting factor, given that the strong annual recruitment near TZ-HB presumably sends larvae to the lower estuary. Comparing the viability of larvae in different reaches of the estuary is an important next step.

Surprisingly, the high reproductive potential found for experimental Martha’s Vineyard oysters at the IRV river site was for 2017 when the IRV site had experienced average salinities ≤ 5 ppt since February (S1 Table). To our knowledge, this is the first demonstration of advanced gametogenesis for *C*. *virginica* under extreme low salinity (< 5 ppt), contradicting previously reported thresholds of 5–40 ppt and “optimal” ranges of 14–28 ppt necessary for growth and reproduction [25]. La Peyre et al. [59] suggested an optimal salinity range of 9–13 ppt in Louisiana oysters. Survival was possible below this range but was accompanied by reduced growth and gametogenesis [59,60].

The relative performance results reported here are especially informative because they apply to a single cohort from mesohaline broodstock that experienced uniform treatment until they were outplanted to different sites at age 3 weeks (3.4 ± 0.1 mm shell length; see Methods). Thus, the spatial variation in performance observed here is not likely due to local adaptation or epigenetic factors (e.g., maternal effects). If the environment experienced by the wild Martha’s Vineyard broodstock or their cultured larvae was the dominant factor determining age-2 gonad maturation across environments, for example, then we would have expected oysters in Jamaica Bay to have outperformed other sites because it supported fast growth and had average salinity more similar to that of the hatchery cohort parents. Instead, given the uniform genetics represented by the hatchery cohort, the observed spatial variation in gonad maturation is interpreted as resulting from (1) site-specific interactions among environmental factors that affect the overall energy budget and/or triggers for reproduction, or (2) site specific variation in the timing (rather than extent) of gametogenesis. Because we were not able to analyze a time series, the hypothesis that Jamaica Bay oysters were already spawned-out in late July could not be rejected. Future research on reproduction should include a time series for sites along the salinity gradient, and might profitably be applied to transplanted TZ-HB wild oysters to specifically test their capabilities across environments.

Before discussing the broader HRE restoration potential afforded by this super-hero TZ-HB population, we critically examine these measures of oyster performance, identify caveats, and put them into context based on the literature. Our inferences are primarily based on the experimental hatchery cohort produced from wild mesohaline parents. We consider the 5-site comparison between caged wild-set spat and hatchery cohorts to be of interest because it contrasts hatchery-produced spat performance against the most relevant control population for assessing the efficacy of population supplementation methods utilizing hatchery production. This type of comparison to approximately same-age wild-set oysters has rarely been reported as far as we know (but see [61]). Nonetheless, the comparison is unreplicated at this point and therefore anecdotal.

### Mortality factors

Extreme exposure to low salinity has shown negative impacts on survival at all life stages [29,62,63]. However, the seasonal timing and rate of change are critical factors in determining the range of tolerance. Where fresh water releases have been studied, their direct effects have been relatively benign to oysters during winter and spring (<5 ppt for 3 weeks), but can be lethal in the summer months when temperatures are elevated [59,63,64]. Previous work in the HRE has attributed mortality of experimental outplants to the synergistic effects of thermal and osmotic stress, suggesting that salinities ≤ 3 ppt in the summer months are lethal to the eastern oyster [29,65]. However, the extreme low was observed in August which coincides with the highest temperatures and summer mortality is often reported when stress accompanies or directly follows peak spawning activity [66]. Thus, the timing of low salinity events may explain differences in survival between studies. For example, the most severe low salinity stress in this study occurred during the second year (2017) of monitoring when mean monthly salinity ranging from 2–4 ppt through July 2017. However, peak temperatures were not observed until August, perhaps contributing to high interval monthly survival at IRV in the summer of 2017 despite very low salinity (S1 Fig).

In this study high mortality was observed at the two most upstream sites (PM and IRV) in April 2016 following a period of prolonged low salinity (≤ 3 ppt), but at the same time HH had high survival under nearly identical salinity conditions. The extreme over-winter mortality (100%) observed at PM (2016) and HH (2017) may be partially dependent on factors other than just low salinity. PM and HH were the only sites in shallow water where the possibility of icing and periods of prolonged aerial exposure during low tides may have contributed to the high winter mortality rate. It should be noted that none of the cage deployments in this study are particularly representative of potentially more favorable deeper subtidal habitats, but they offer a logistically tractable means of comparing oyster performance across the estuary.

Salinity in New York Harbor remained within the optimal range for oysters throughout the study period, suggesting that other environmental conditions were lowering survivorship. Prevalence of oyster predators could have had a major role in mortality at harbor and Jamaica Bay sites. During the first few months after outplant, a heavy presence of the predatory worm *Stylochus ellipticus* was observed and was a likely contributor to the high mortality observed at New York Harbor sites. Even at low densities, *S*. *ellipticus* can devastate recently metamorphosed spat [67]. A high occurrence of *S*. *ellipticus* at PGB during 2015 (first fall) could also account for the low oyster survival compared to the following years when *S*. *ellipticus* was less prevalent. The boring sponge *Cliona celata* was prevalent during summer 2017 monitoring at harbor and Jamaica Bay sites. Most impacted was KCC, which partially explains low survival rates compared to previous years. Heavy boring sponge infestation at KCC may also explain the low condition and gonad maturation index rankings observed [68]. The presence of *C*. *celata* causes shell damage resulting in a diversion of energy from growth and reproduction to shell repair [69]. Thus, in addition to the well-documented gradient in predators and pathogens causing more top-down population regulation at higher salinities [70,71], sublethal predation pressures also can reduce reproductive output by inhibiting gametogenesis [72]. The rapid growth rates observed at KCC in the first year (through 2016) can make the area seem attractive for oyster restoration, but would need to be combined with environmental factors that inhibit some of these top down effects. Unlike mid-Atlantic populations of eastern oyster where intertidal habitat is hypothesized to offer a refuge from predation at higher salinities [73], higher latitude populations are subject to winter freezing if exposed by the tide. Given that the population ecology of *C*. *virginica* has mostly been studied south of the latitudinal freeze zone, it is an open question how far toward the ocean temperate estuarine oyster populations can sustain populations.

In suboptimal environments, growth and reproduction may be inhibited by increased sedimentation and pollution rates. New York Harbor and the lower Hudson River along Manhattan experience the estuarine turbidity maximum [74], potentially generating increased sedimentation rates that can lower growth and survival. Increased turbidity may also result in resuspension or influx of persistent organic pollutants from both current and historic sources in the HRE [75,76] which can amplify negative effects of turbid environments. Except for the fact that the western Hudson shore gets much more sedimentation than the eastern shore along Manhattan [77], sedimentation rates are highly variable both temporally and spatially within this region [78], making correlative links impossible in this study. Anecdotally, we observed more siltation of oyster cages at some sites than others (e.g. Red Hook, Irvington), perhaps contributing to intra-regional performance variation.

An intriguing pattern of age-2 late summer oyster mortality reported by Levinton et al. [65] for harbor and Jamaica Bay experimental sites was not observed here. Their explanatory hypothesis of cumulative stress in the urban estuary may hold for the selectively-bred aquaculture strain they studied, but no such effects are detectable in the results of this study. The fact that their unpolluted control site also showed elevated age-2 mortality in September is consistent with intrinsic factors affecting their experimental cohort.

### Growth rate

Bivalve growth is significantly affected by the environment they are exposed to and as sessile organisms, shifting energy allocation from growth to maintenance can help to reduce the impacts of environmental stress [79]. Salinity, temperature, acidification, pollutants, disease and food availability are among some of the commonly encountered stressors [25,80]. While oysters can tolerate a wide range of salinities (5–40 ppt [81]), the best growth and survival has been observed at salinities > 10 ppt and relatively low growth attributed to reduced feeding was observed at salinities ≤ 5 ppt [63,64,82,83]. In this study we observed high growth rates at river sites when salinity was 6–10 ppt, but when salinity was < 5 ppt (June-July 2017) growth at IRV was negligible, suggesting a threshold of ~ 6 ppt for growth of hatchery outplants in the HRE. The fastest growth in the HRE was observed in Jamaica Bay where salinities were high and associated with relatively high chlorophyll-*a* (Fig 2). Contrary to what is expected based solely on salinity patterns, the lowest growth was observed in New York Harbor where salinity remained within the optimal range throughout the experimental period. This suggests that other environmental factors may play a more important role in shaping relative performance of eastern oysters across HRE regions, a pattern also seen by Dunn et al. [84] in North Carolina estuaries.

A spike in growth rate was observed at RED in July 2017, however oyster size distributions did not increase between June and July sampling as would be expected based on the observed growth rate (S2 and S3 Figs), suggesting size specific mortality that may be partially explained by predation pressures and sedimentation. The extent of this bias was surprising because for all other months and sites, some with intense sedimentation and abundant predators, size biased morality was not evident based on size distributions and variance in length.

In the first month of cage deployment for wild-set HH and SV oysters, a 26–29% increase in counts suggests that hatchery cohort oysters at river sites, and perhaps elsewhere, became contaminated with an unknown proportion of wild-set oysters. In October 2015, when the average spat size ranged 13.5–21.0 mm among sites, we started culling spat ≤10 mm shell length from hatchery bags to exclude them from performance measures, but this was likely an incomplete solution. There was no obvious mode of smaller spat in size distributions so we were unable to quantify the magnitude of this contamination and test for a resulting bias in growth rate. Contaminating wild-set spat was more easily identified and prevented in 2016, but the initial 2015 contamination could have had lasting effects for the HH and IRV sites where wild recruitment was substantial.

### Disease

The optimal salinity range for two of the most devastating oyster pathogens, *Perkinsus marinus* (dermo) and *Haplosporidum nelsoni* (MSX), coincides with the favorable salinity range reported for oysters (> 12 ppt [83,85]). These pathogens have previously been associated with high oyster mortality in New York Harbor and Jamaica Bay [29,65]. Although no indication of dermo related morality was observed during this study, we are not able to exclude disease as a factor affecting oyster performance. First, no testing for MSX was conducted. Second, sublethal effects of infection may have contributed to relatively low performance in the harbor where we observed the highest prevalence of dermo. Sublethal infections can reduce growth, reproduction, and physiological condition due to disruptions in energy allocation towards tissue repair and maintenance costs [79,86] and can have lasting effects on population structure [29,87].

Under high disease pressure, moderate to high salinity reefs have little hope of long term survival without continuous replenishment, either natural or via hatchery-based supplementation [88]. In southern geographical regions there appears to be some purging of pathogen pressure when low salinity freshets (< 10 ppt generated by high precipitation or fresh water releases) occur in the winter and early spring, abating disease infections by reducing salinity outside of the pathogen tolerance range and thereby reducing disease body burden without high temperatures exacerbating osmotic stress [59,82,88,89]. Salinity variation during this study and in 2008–2009 [29] suggest that HRE harbor and lower Manhattan sites will infrequently experience these low salinity levels, but much of the Hudson River to the north could enjoy some protection from disease via this mechanism.

### Gametogenesis and condition index

Gametogenesis in adults (fecundity) and successful settlement of new recruits are essential to long term population success for both restored and natural reefs. While gametogenic cycles are driven by temperature and food availability [90], environmental stress factors potentially limiting gametogenesis in the eastern oyster including low salinity [60,64] and disease [87,91]. Loosanoff [64] observed reduced gametogenic activity at 5 ppt and complete inhibition at 3 ppt in Long Island Sound oysters. Butler [92] observed delayed gametogenesis until salinity rose to ≥ 6 ppt. In more recent reports, mature gonads have been observed at salinities as low as 6 ppt, but at 3 ppt gamete resorption was evident [93]. Comparisons made across the salinity gradient in Pamlico Sound showed greater size-specific fecundity inshore (average salinity: 18 ppt) by a factor of 2–3, but the outer bay reefs (average salinity: 22 ppt) nonetheless had greater demographic impact due to their higher oyster abundance [72]. This regional fecundity difference, despite smaller oyster sizes inshore, was attributed to parasite and pathogen stressors at higher salinities. We observed mature gonads in this study at salinities < 5 ppt. Mean salinity in the 8 weeks leading up to sampling in July 2017 was 3.1 ± 0.15 ppt at IRV. Daily averages in June were all <5 ppt and only 3 days in July had averages above 5 (S1 Table), conditions previously reported to inhibit reproduction [64,92,94]. This demonstrates that oysters of mesohaline parentage are not only capable of surviving extreme low salinity, but that they are capable of gonad maturation as well.

At New York Harbor and Jamaica Bay sites the proportion of individuals with developing gametes was relatively low, but signs of active gametogenesis were evident in some individuals at all sites. For example, At Jamaica Bay site KCC both gametogenesis and condition index were low in 2017 with only 21% having differentiated gametes and none had mature gonads. The infestation of boring sponges at KCC may have contributed to this poor performance [68]. Although a single sampling timepoint for gametogenesis potentially misses early or late maturation, previous work in Jamaica Bay and New York Harbor has shown experimental outplants (a domesticated aquaculture strain) to have differentiated gametes throughout most of June–August [65]. However, across two experiments in different years there was substantial variation in the proportion of oysters having differentiated gametes [65]. Interestingly, differentiated gametes also were observed in a high proportion of age-1 oysters by Levinton et al. [65] but their methods make it difficult to relate that to spawning condition. Spawning season for Long Island Sound, nearby at the same latitude, also is known to extend through August [95]. Thus, it is possible that the single sample in July in this study missed an earlier or later peak in activity at harbor and Jamaica Bay sites. Additional surveys of gonad maturation are needed that can account for possibly distinct phenology in different regions of the HRE.

### Recruitment

The consistent annual spat recruitment observed at HH and IRV indicates that a wild remnant population of oysters is reproducing in the HRE. Two hypotheses warrant further investigation. First, that undocumented oyster populations downriver, where salinities are moderate to high, are reproducing with upstream larval transport supplying the TZ-HB area with an annual recruitment class. No contemporary subtidal oyster surveys have been conducted south of the TZ-HB area (but see [96]. Second, the adult oysters found at TZ-HB are successfully spawning and producing larvae under low salinity conditions. Both scenarios suggest that the larvae are capable of tolerating salinities ≤ 10 ppt during months when good recruitment was observed. The high prevalence of gonad maturity at IRV found for hatchery-produced oysters from mesohaline parents implies that TZ-HB wild oysters also are capable of gametogenesis under extreme low salinity (< 5 ppt). Both scenarios beg the question of larval fate south of the TZ-HB area, given the sporadic and sparse spat recruitment observed in the mesohaline portion of the HRE [31,32,97].

Previous work in Jamaica Bay has documented an absence of oyster spat recruitment [29,32,65] despite suitable conditions for juvenile growth and survivorship. This could result from low numbers of larvae being produced in or entering Jamaica Bay, or be an indication that the waters in Jamaica Bay are not suitable for larval survival and settlement. Jamaica Bay has been reported to experience periods of hypoxia [65] and has high sewage discharge pollution [98], potentially reducing larval survivorship below levels that could support detectable recruitment. Studies of spat recruitment in other regions have found a positive correlation with salinity [84,99,100]. There are many possible mechanisms generating this pattern, such as a gradient in oyster population density or reproduction, but it still stands as a contrast to the situation in the HRE.

### Implications for restoring a sustainable oyster metapopulation to the HRE

Based on spat recruitment, this study establishes the Tappan Zee–Haverstraw Bay oyster population as a robustly reproductive remnant wild population in the HRE. Among the three HRE regions studied (river, harbor, Jamaica Bay), the river north of Manhattan supported the best oyster performance in terms of wild spat recruitment and July gametogenesis. River sites were not as good as Jamaica Bay for juvenile survival and growth, but the fully subtidal cages at river sites yielded sufficient survival and growth to reproduce at age 2. Because this study only evaluated oyster performance for 2 years, and did not include tests on larvae, the question remains as to whether Jamaica Bay can support an oyster population. Furthermore, given the larval supply presumably produced by TZ-HB oysters, why is there no spat recruitment downstream? We offer two non-mutually exclusive hypotheses for future testing. First, larvae from the TZ-HB population may suffer severe mortality in downstream reaches of the Hudson River due to low water quality, diminishing their ability to found and support downstream subpopulations [101], and possibly compounded by low advection supply to and/or retention within Jamaica Bay [102]. Alternatively, suitable subtidal habitat for larval settlement may be almost entirely lacking downstream of TZ-HB. Testing these hypotheses will be a critical step forward for scaling-up oyster restoration efforts in the HRE.

Distance of the TZ-HB population from the harbor may be to its benefit, but it’s isolation also puts the HRE remnant wild population at risk. With a higher frequency and amplitude of storm events predicted under climate change [103], it is risky having the only regularly reproducing population restricted to low salinity reaches of the river where heavy rainfall events can rapidly drop the already low salinity. When these events coincide with warm temperatures they can be devastating, at least for shallow subtidal oysters [29]. A more resilient population would include reproductive populations elsewhere in the estuary where larval connectivity could tie the metapopulation together both demographically and evolutionarily.

While salinity is often the focus when assessing oyster population distribution, approaching this as a singular effect neglects important interacting factors along a given salinity gradient. Similarly, an exclusive focus on juvenile performance without considering spat recruitment or reproduction would have led to different conclusions here. If our results for the experimental hatchery cohort are representative of performance variation we would see from transplanted wild TZ-HB oysters, then it suggests a different restoration strategy than has recently been proposed and studied [8,104]. Instead of using hatcheries to produce seed for restoration plantings in the HRE, perhaps a more sustainable and successful source of seed is the TZ-HB population. To take advantage of strong and consistent spat recruitment in the TZ-HB area, oyster cultch (settlement substrate) could be deployed in late summer and retrieved later that fall for transplantation to subtidal sites with cleared or hardened benthos. Habitat suitability in the East River, along the New Jersey side of the Hudson River, and in Raritan Bay in the lower HRE also need to be evaluated to realize a sufficient network of reproductive subpopulations connected by larval dispersal. Unfortunately, effective larval dispersal pathways are a continuing information gap for the HRE.

## Conclusions

The historic oyster reef footprint of 18^th^ century New York is never likely to be restored, but viability of the Hudson/Raritan Estuary oyster population depends on restoring some metapopulation structure. Here, we furthered this goal by identifying spatial variation in oyster performance in different regions of the estuary, identifying not-so-surprising performance deficits at harbor sites and surprisingly strong performance at low salinity river sites. The strong oyster performance in Jamaica Bay is consistent with previous findings. The combined pressures of disease, predation and pollution in the lower portion of the HRE, where salinities would otherwise suggest ideal growing conditions for oysters, have resulted in the isolation of a remnant wild eastern oyster population in the low salinity reaches of the Hudson River. The results of this study support and build upon that presented by Levinton et al. [29] who first suggested that the TZ-HB population is ecologically valuable and in danger from climate change threats. With metapopulation principles guiding restoration planning [16] it becomes clear that not every reef needs to be in ideal habitat, as long as conditions support the full life cycle, including larval dispersal. Because discovery of robust TZ-HB recruitment offers the possibility of transplant-based restoration, future studies comparing performance should not rely solely on outplants of hatchery-produced cohorts from elsewhere. It is possible that the TZ-HB population is locally adapted and will not perform well at higher salinities. Rigorous tests are needed for the relative performance of hatchery-based seed oysters versus TZ-HB wild-set spat in all regions of the HRE. For example, the capacity of different HRE regions to support oyster reproduction needs further testing to take into account within-estuary variation in seasonality as well as compare fecundity in wild TZ-HB vs. hatchery-produced oysters. Ultimately, self-sustaining oyster populations will only persist if every critical component of the life cycle–growth, reproduction, larval survival and settlement–is supported in a metapopulation that has demographic resilience and sufficient genetic diversity to maintain adaptive capacity under environmental change.

## Supporting information

S1 Fig Plots for each site showing interval mortality between each sampling point (bars) and mean monthly salinity (line plot). In July 2016 a negative mortality (indicated with *) at IRV and HH suggests that local recruitment is increasing the total spat count at each site.
(TIF)

S2 Fig Length distributions of all oysters remaining at RED in June 2017 (N = 95) and July 2017 (N = 51). (TIF)

S3 Fig Comparion of observed June length distrbutions (yellow) with July length predictions (pink). And July predictions (pink) compared with observed length distributions in July (green). Length predictions were made using the mean growth rate (16.3 mm / month) between the two sampling points and assumes no mortality. Predictions show larger individuals (right end of the tail) than actually observed and there were more smaller oysters observed than predicted (left end of the tail) suggesting some size related mortality causing artifical inflation of the growth rate. The growth rate used was pre-normalization to reflect the entired 6-week growing period between measurements.
(TIF)

S1 Table Observed salinity extremes at Irvington (IRV) identifying the number and proportion of days < 5 ppt, mean monthly salinity and respective mean temperature for each month over the monitoring period (N = 706 days). (DOCX)
