# Correction: Restoring oysters to urban estuaries: Redefining habitat quality for eastern oyster performance near New York City

**DOI:** 10.1371/journal.pone.0218535

**Published:** 2019-06-13

**Authors:** Katherine McFarland, Matthew P. Hare

The following information is missing from the Funding statement: This study was also supported by the New York Sea Grant project R/XG-22.
