# Supplementary figures and images for: Restoring oysters to urban estuaries: Redefining habitat quality for eastern oyster performance near New York City
Source: PLoS One. 2018 Nov 16;13(11):e0207368. doi: 10.1371/journal.pone.0207368 (PMC6239315; doi:10.1371/journal.pone.0207368)

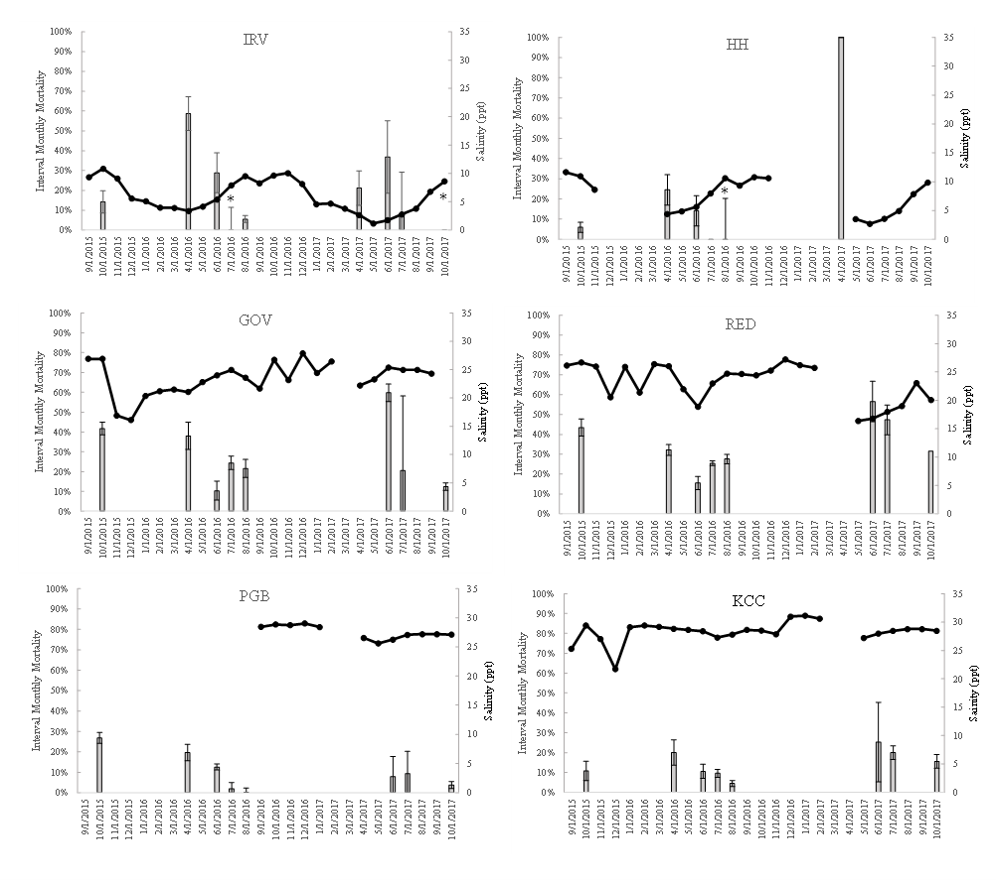

Supplement: S1 Fig — In July 2016 a negative mortality (indicated with *) at IRV and HH suggests that local recruitment is increasing the total spat count at each site. (TIF) [file pone.0207368.s001.tif]

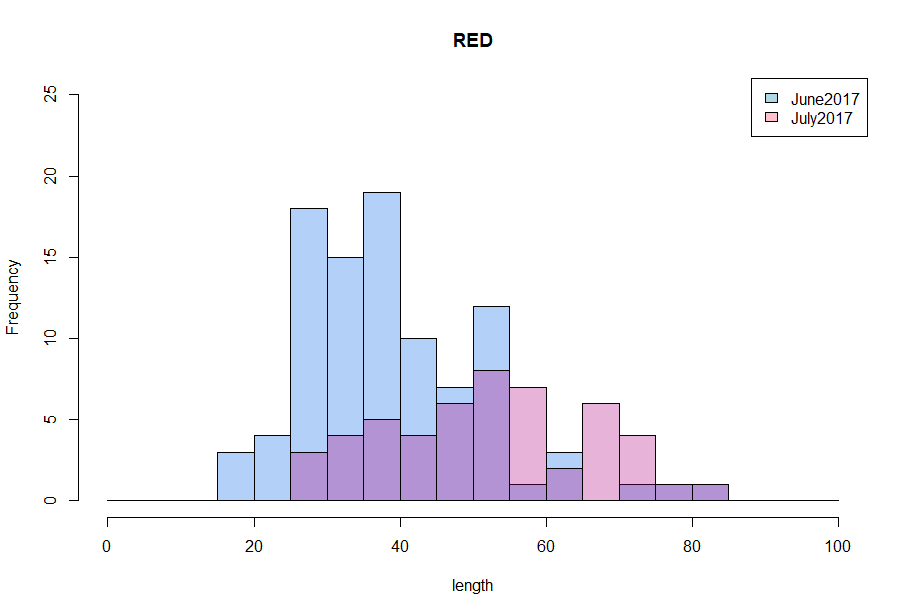

Supplement: S2 Fig — (TIF) [file pone.0207368.s002.tif]

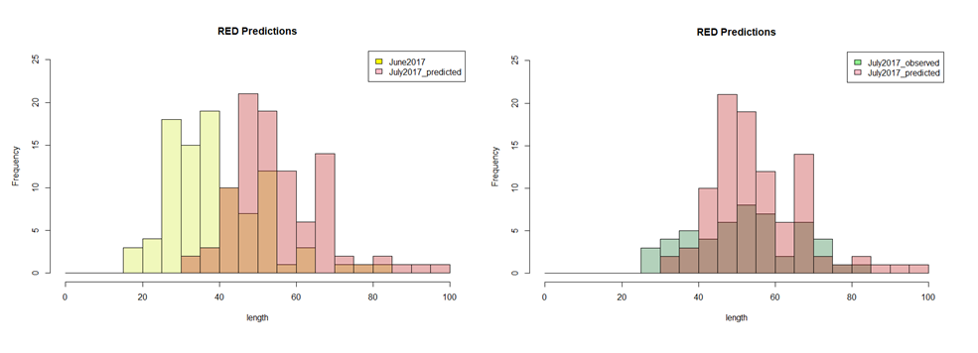

Supplement: S3 Fig — And July predictions (pink) compared with observed length distributions in July (green). Length predictions were made using the mean growth rate (16.3 mm / month) between the two sampling points and assumes no mortality. Predictions show larger individuals (right end of the tail) than actually observed and there were more smaller oysters observed than predicted (left end of the tail) suggesting some size related mortality causing artifical inflation of the growth rate. The growth rate used was pre-normalization to reflect the entired 6-week growing period between measurements. (TIF) [file pone.0207368.s003.tif]
