# Supplementary material for: Restoring oysters to urban estuaries: Redefining habitat quality for eastern oyster performance near New York City
Source: PLoS One. 2018 Nov 16;13(11):e0207368. doi: 10.1371/journal.pone.0207368 (PMC6239315; doi:10.1371/journal.pone.0207368)
Supplement: S1 Table — (DOCX) [file pone.0207368.s004.docx]

| **S1 Table. Observed salinity extremes at Irvington (IRV) identifying the number and proportion of days < 5 ppt, mean monthly salinity and respective mean temperature for each month over the monitoring period (N = 706 days).** | | | | | |
| --- | --- | --- | --- | --- | --- |
|  | **< 5 ppt (days)** | **< 5 ppt (%)** | **Average Salinity** (± SE) | **Average Temperature** (± SE) |  |
| **2015** |  |  |  |  |  |
| September | 0 | 0.00% | 10.82 (± 0.15) | 24.83 (± 0.27) |  |
| October | 0 | 0.00% | 9.08 (±0.20) | 17.58 (± 0.37) |  |
| November | 10 | 33.33% | 5.53 (± 0.19) | 13.00 (± 0.34) |  |
| December | 15 | 48.39% | 5.08 (± 0.39) | 9.30 (± 0.12) |  |
| **2016** |  |  |  |  |  |
| January | 22 | 70.97% | 3.95 (± 0.34) | 3.57 (± 0.30) |  |
| February | 21 | 72.41% | 3.90 (± 0.40) | 2.15 (± 0.22) |  |
| March | 27 | 87.10% | 3.38 (± 0.25) | 5.93 (± 0.31) |  |
| April | 17 | 56.67% | 4.14 (± 0.330 | 10.52 (± 0.40) |  |
| May | 12 | 38.71% | 5.41 (± 0.28) | 15.39 (± 0.41) |  |
| June | 1 | 3.33% | 7.94 (± 0.35) | 21.72 (± 0.20) |  |
| July | 0 | 0.00% | 9.54 (± 0.09) | 26.56 (± 0.23) |  |
| August | 0 | 0.00% | 8.63 (± 0.15) | 27.64 (± 0.16) |  |
| September | 0 | 0.00% | 9.85 (± 0.22) | 23.49 (± 0.24) |  |
| October | 0 | 0.00% | 10.05 (± 0.12) | 17.51 (± 0.49) |  |
| November | 0 | 0.00% | 9.12 (± 0.12) | 11.00 (± 0.36) |  |
| December | 20 | 63.50% | 4.56 (± 0.20) | 5.44 (± 0.37) |  |
| **2017** |  |  |  |  |  |
| January | 16 | 66.67% | 4.67 (± 0.21) | 2.84 (± 0.18) |  |
| February | 26 | 92.86% | 3.77 (± 0.16) | 2.86 (± 0.29) |  |
| March | 28 | 90.32% | 2.65 (± 0.27) | 3.91 (± 0.25) |  |
| April | 30 | 100% | 1.15 (± 0.19) | 10.06 (± 0.48) |  |
| May | 30 | 96.77% | 1.73 (± 0.26) | 16.04 (± 0.29) |  |
| June | 30 | 100% | 2.78 (± 0.12) | 21.77 (± 0.41) |  |
| July | 28 | 90.32% | 4.10 (± 0.16) | 25.68 (± 0.20) |  |
| August | 3 | 9.68% | 6.59 (± 0.20) | 25.28 (± 0.17) |  |
| September | 0 | 0% | 8.61 (± 0.15) | 22.31 (± 0.18) |  |
|  |  |  |  |  |  |
| **Total** | **336** | **42.91%** | **5.97 (± 0.11)** | **15.21 (± 0.31)** |  |
